# Supplementary material for: N-acetylation of α-synuclein enhances synaptic vesicle clustering mediated by α-synuclein and lysophosphatidylcholine
Source: eLife. 2024 Dec 27;13:RP97228. doi: 10.7554/eLife.97228 (PMC11677226; doi:10.7554/eLife.97228)
Supplement: Supplementary file 1. [file elife-97228-supp1.docx]

**Supplementary File 1a** Identified cross-linked peptides of sample 1 between ^15^N-Ac-α-syn and ^14^N-Ac-α-syn in LPC (protein:lipid = 1:50, mol:mol).

| **Score** | **Δ (ppm)** | **Res of Pr 1*** | **Sequence of Pr 1*** | **Res of Pr2*** | **Sequence of Pr 2*** | **Mass (Da)** |
| --- | --- | --- | --- | --- | --- | --- |
| 1.43E-20 | -0.67 | 12 | A^11^KEGVVAAAEK^21^ | 23 | T^22^KQGVAEAAGK^32^ | 2268.2324 |
| 5.94E-14 | 0.65 | 32 | Q^24^GVAEAAGKTK^34^ | 10 | G^9^LSKAK^12^ | 1799.0151 |
| 8.04E-13 | -0.04 | 34 | T^33^KEGVLYVGSK^43^ | 23 | T^22^KQGVAEAAGK^32^ | 2376.2899 |
| 2.17E-12 | -1.77 | 32 | T^22^KQGVAEAAGKTK^34^ | 23 | T^22^KQGVAEAAGK^32^ | 2484.3546 |
| 5.89E-12 | 0.46 | 45 | T^44^KEGVVHGVATVAEK^58^ | 12 | T^22^KQGVAEAAGK^32^ | 2733.4911 |
| 1.33E-11 | 0.22 | 12 | A^11^KEGVVAAAEK^21^ | 97 | K^97^DQLGK^102^ | 1897.0519 |
| 2.22E-11 | -2.07 | 58 | T^44^KEGVVHGVATVAEKTK^60^ | 10 | G^9^LSKAK^12^ | 2493.4165 |
| 1.49E-10 | -2.77 | 23 | T^22^KQGVAEAAGK^32^ | 97 | K^97^DQLGK^102^ | 1884.0315 |
| 2.94E-10 | -1.25 | 34 | T^33^KEGVLYVGSK^43^ | 12 | A^11^KEGVVAAAEK^21^ | 2389.3103 |
| 1.42E-08 | -1.10 | 21 | E^13^GVVAAAEKTK^23^ | 10 | G^9^LSKAK^12^ | 1842.0461 |
| 2.07E-08 | -0.67 | 58 | E^46^GVVHGVATVAEKTKEQVTNVGGAVVTGVTAVAQK^80^ | 12 | G^7^LSKAKEGVVAAAEK^21^ | 5027.7505 |
| 3.43E-08 | -2.37 | 43 | T^33^KEGVLYVGSKTK^45^ | 10 | G^9^LSKAK^12^ | 2149.2357 |
| 5.39E-07 | 2.62 | 43 | T^33^KEGVLYVGSKTK^45^ | 43 | T^33^KEGVLYVGSKTK^45^ | 2955.6531 |

Δ: mass error for crosslink assignments; Res: crosslinked residue (sequence number) within corresponding proteins; Pr: protein; Underlined K in sequence is the cross-linked lysine.

*The crosslinked pair of residues were from two individual Ac-α-syn: ^14^N-Ac-α-syn and ^14^N-Ac-α-syn, ^14^N-Ac-α-syn and ^15^N-Ac-α-syn, ^15^N-Ac-α-syn and ^14^N-Ac-α-syn, or ^15^N-Ac-α-syn and ^15^N-Ac-α-syn. Detailed data processing procedure could be referred to Methods session.

**Supplementary File 1b** Identified cross-linked peptides of sample 2 between ^15^N-Ac-α-syn and ^14^N-Ac-α-syn in LPC (protein:lipid = 1:50, mol:mol).

| **Score** | **Δ (ppm)** | **Res of Pr 1*** | **Sequence of Pr 1*** | **Res of Pr2*** | **Sequence of Pr 2*** | **Mass (Da)** |
| --- | --- | --- | --- | --- | --- | --- |
| 2.56E-17 | -0.42 | 45 | T^44^KEGVVHGVATVAEK^58^ | 12 | A^11^KEGVVAAAEK^21^ | 2733.4911 |
| 3.92E-15 | 0.45 | 12 | A^11^KEGVVAAAEK^21^ | 23 | T^22^KQGVAEAAGK^32^ | 2268.2324 |
| 3.41E-14 | 0.97 | 34 | T^33^KEGVLYVGSK^43^ | 23 | T^22^KQGVAEAAGK^32^ | 2376.2899 |
| 2.82E-13 | 0.45 | 32 | Q^24^GVAEAAGKTK^34^ | 10 | G^7^LSKAK^10^ | 1799.0151 |
| 6.88E-13 | 0.59 | 43 | T^33^KEGVLYVGSKTK^45^ | 12 | A^11^KEGVVAAAEK^21^ | 2618.4529 |
| 4.51E-12 | 1.04 | 12 | A^11^KEGVVAAAEK^21^ | 97 | K^97^DQLGK^102^ | 1897.0519 |
| 2.59E-11 | -2.90 | 23 | T^22^KQGVAEAAGK^32^ | 97 | K^97^DQLGK^102^ | 1884.0315 |
| 1.28E-10 | 0.36 | 32 | Q^24^GVAEAAGKTK^34^ | 97 | K^97^DQLGK^102^ | 1884.0315 |
| 1.07E-09 | -1.42 | 21 | A^11^KEGVVAAAEKTK^23^ | 12 | A^11^KEGVVAAAEK^21^ | 1965.9903 |
| 2.81E-09 | 2.82 | 21 | E^13^GVVAAAEKTK^23^ | 10 | G^7^LSKAK^10^ | 2510.3954 |
| 7.27E-08 | 0.30 | 43 | T^33^KEGVLYVGSKTK^45^ | 97 | K^97^DQLGK^102^ | 1842.0461 |

Δ: mass error for crosslink assignments; Res: crosslinked residue (sequence number) within corresponding proteins; Pr: protein; Underlined K in sequence is the cross-linked lysine.

*The crosslinked pair of residues were from two individual Ac-α-syn: ^14^N-Ac-α-syn and ^14^N-Ac-α-syn, ^14^N-Ac-α-syn and ^15^N-Ac-α-syn, ^15^N-Ac-α-syn and ^14^N-Ac-α-syn, or ^15^N-Ac-α-syn and ^15^N-Ac-α-syn. Detailed data processing procedure could be referred to Methods session.

**Supplementary File 1c** Identified cross-linked peptides of sample 3 between ^15^N-Ac-α-syn and ^14^N-Ac-α-syn in LPC (protein:lipid = 1:50, mol:mol).

| **Score** | **Δ (ppm)** | **Res of Pr 1*** | **Sequence of Pr 1*** | **Res of Pr2*** | **Sequence of Pr 2*** | **Mass (Da)** |
| --- | --- | --- | --- | --- | --- | --- |
| 3.49E-15 | 0.21 | 12 | A^11^KEGVVAAAEK^21^ | 23 | T^22^KQGVAEAAGK^32^ | 2268.2324 |
| 1.32E-13 | 3.78 | 60 | T^59^KEQVTNVGGAVVTGVTAVAQK^80^ | 34 | T^33^KEGVLYVGSK^43^ | 3473.8980 |
| 4.26E-13 | 2.34 | 34 | T^33^KEGVLYVGSK^43^ | 23 | T^22^KQGVAEAAGK^32^ | 2376.2899 |
| 1.14E-12 | 0.67 | 12 | A^11^KEGVVAAAEK^21^ | 97 | K^97^DQLGK^102^ | 1897.0519 |
| 2.32E-12 | 1.24 | 45 | T^44^KEGVVHGVATVAEK^58^ | 23 | T^22^KQGVAEAAGK^32^ | 2720.4707 |
| 5.94E-12 | 1.20 | 96 | T^81^VEGAGSIAAATGFVKK^97^ | 12 | A^11^KEGVVAAAEK^21^ | 2815.5330 |
| 1.07E-10 | 0.52 | 43 | T^33^KEGVLYVGSKTK^45^ | 12 | A^11^KEGVVAAAEK^21^ | 2618.4529 |
| 5.04E-09 | 0.91 | 21 | A^11^KEGVVAAAEKTK^23^ | 97 | K^97^DQLGK^102^ | 2126.1946 |
| 2.29E-08 | 1.40 | 96 | T^91^VEGAGSIAAATGFVKK^97^ | 23 | T^22^KQGVAEAAGK^32^ | 2802.5126 |
| 1.52E-07 | 1.15 | 43 | T^33^KEGVLYVGSKTK^45^ | 10 | G^7^LSKAK^10^ | 2149.2357 |
| 2.52E-07 | -3.62 | 34 | T^33^KEGVLYVGSK^43^ | 12 | A^11^KEGVVAAAEK^21^ | 2389.3103 |
| 5.32E-07 | -2.34 | 60 | T^59^KEQVTNVGGAVVTGVTAVAQK^80^ | 21 | E^13^GVVAAAEKTK^23^ | 3395.8510 |
| 7.21E-07 | 0.58 | 12 | A^11^KEGVVAAAEK^21^ | 12 | A^11^KEGVVAAAEK^21^ | 2281.2528 |
| 7.33E-07 | 2.58 | 32 | T^22^KQGVAEAAGKTK^34^ | 23 | T^22^KQGVAEAAGK^32^ | 2484.3546 |
| 8.89E-07 | 0.99 | 21 | E^13^GVVAAAEKTK^23^ | 10 | G^7^LSKAK^10^ | 1842.0461 |

Δ: mass error for crosslink assignments; Res: crosslinked residue (sequence number) within corresponding proteins; Pr: protein; Underlined K in sequence is the cross-linked lysine.

*The crosslinked pair of residues were from two individual Ac-α-syn: ^14^N-Ac-α-syn and ^14^N-Ac-α-syn, ^14^N-Ac-α-syn and ^15^N-Ac-α-syn, ^15^N-Ac-α-syn and ^14^N-Ac-α-syn, or ^15^N-Ac-α-syn and ^15^N-Ac-α-syn. Detailed data processing procedure could be referred to Methods session.

**Supplementary File 1d** Identified cross-linked peptides of sample 1 between ^15^N-Ac-α-syn and ^14^N-Ac-α-syn in DOPS (protein:lipid = 1:50, mol:mol).

| **Score** | **Δ (ppm)** | **Res of Pr 1*** | **Sequence of Pr 1*** | **Res of Pr2*** | **Sequence of Pr 2*** | **Mass (Da)** |
| --- | --- | --- | --- | --- | --- | --- |
| 2.08E-16 | 0.45 | 32 | Q^24^GVAEAAGKTK^34^ | 10 | G^7^LSKAK^10^ | 1799.0151 |
| 2.67E-14 | 2.65 | 45 | T^44^KEGVVHGVATVAEK^58^ | 23 | T^22^KQGVAEAAGK^32^ | 2720.4707 |
| 4.22E-14 | -2.67 | 45 | T^44^KEGVVHGVATVAEK^58^ | 12 | A^11^KEGVVAAAEK^21^ | 2733.4911 |
| 1.76E-12 | -0.66 | 12 | A^11^KEGVVAAAEK^21^ | 23 | T^22^KQGVAEAAGK^32^ | 2268.2324 |
| 1.92E-09 | -0.87 | 21 | E^13^GVVAAAEKTK^23^ | 23 | T^22^KQGVAEAAGK^32^ | 2298.2430 |
| 8.17E-09 | 1.29 | 21 | A^11^KEGVVAAAEKTK^23^ | 10 | G^7^LSKAK^10^ | 2041.1782 |

Δ: mass error for crosslink assignments; Res: crosslinked residue (sequence number) within corresponding proteins; Pr: protein; Underlined K in sequence is the cross-linked lysine.

*The crosslinked pair of residues were from two individual Ac-α-syn: ^14^N-Ac-α-syn and ^14^N-Ac-α-syn, ^14^N-Ac-α-syn and ^15^N-Ac-α-syn, ^15^N-Ac-α-syn and ^14^N-Ac-α-syn, or ^15^N-Ac-α-syn and ^15^N-Ac-α-syn. Detailed data processing procedure could be referred to Methods session.

**Supplementary File 1e** Identified cross-linked peptides of sample 2 between ^15^N-Ac-α-syn and ^14^N-Ac-α-syn in DOPS (protein:lipid = 1:50, mol:mol).

| **Score** | **Δ (ppm)** | **Res of Pr 1*** | **Sequence of Pr 1*** | **Res of Pr2*** | **Sequence of Pr 2*** | **Mass (Da)** |
| --- | --- | --- | --- | --- | --- | --- |
| 3.01E-15 | -0.73 | 43 | T^33^KEGVLYVGSKTK^45^ | 43 | E^35^GVLYVGSKTK^45^ | 2726.5105 |
| 1.71E-11 | 2.06 | 96 | T^81^VEGAGSIAAATGFVKK^97^ | 12 | A^11^KEGVVAAAEK^21^ | 2815.5330 |
| 1.22E-10 | -2.90 | 32 | Q^24^GVAEAAGKTK^34^ | 97 | K^97^DQLGK^102^ | 1884.0315 |
| 3.80E-08 | -0.35 | 96 | T^81^VEGAGSIAAATGFVKK^97^ | 10 | G^7^LSKAK^10^ | 2346.3157 |
| 4.64E-07 | 0.38 | 23 | T^22^KQGVAEAAGK^32^ | 23 | T^22^KQGVAEAAGK^32^ | 2255.2120 |

Δ: mass error for crosslink assignments; Res: crosslinked residue (sequence number) within corresponding proteins; Pr: protein; Underlined K in sequence is the cross-linked lysine.

*The crosslinked pair of residues were from two individual Ac-α-syn: ^14^N-Ac-α-syn and ^14^N-Ac-α-syn, ^14^N-Ac-α-syn and ^15^N-Ac-α-syn, ^15^N-Ac-α-syn and ^14^N-Ac-α-syn, or ^15^N-Ac-α-syn and ^15^N-Ac-α-syn. Detailed data processing procedure could be referred to Methods session.

**Supplementary File 1f** Identified cross-linked peptides of sample 3 between ^15^N-Ac-α-syn and ^14^N-Ac-α-syn in DOPS (protein:lipid = 1:50, mol:mol).

| **Score** | **Δ (ppm)** | **Res of Pr 1*** | **Sequence of Pr 1*** | **Res of Pr2*** | **Sequence of Pr 2*** | **Mass (Da)** |
| --- | --- | --- | --- | --- | --- | --- |
| 4.55E-14 | 0.36 | 32 | Q^24^GVAEAAGKTKEGVLYVGSK^43^ | 21 | A^11^KEGVVAAAEKTK^23^ | 3429.8717 |
| 9.60E-14 | 0.25 | 32 | Q^24^GVAEAAGKTK^34^ | 10 | G^7^LSKAK^10^ | 1799.0151 |
| 1.05E-07 | -1.97 | 45 | T^44^KEGVVHGVATVAEK^58^ | 43 | E^35^GVLYVGSKTK^45^ | 2841.5486 |
| 1.08E-07 | 1.16 | 23 | T^22^KQGVAEAAGK^32^ | 32 | Q^24^GVAEAAGKTK^34^ | 2255.2120 |
| 2.30E-07 | -1.49 | 96 | T^81^VEGAGSIAAATGFVKK^97^ | 12 | A^11^KEGVVAAAEK^21^ | 2815.5330 |
| 5.89E-07 | 3.15 | 6 | M^1^DVFMKGLSK^10^ | 32 | Q^24^GVAEAAGKTK^34^ | 2351.2228 |

Δ: mass error for crosslink assignments; Res: crosslinked residue (sequence number) within corresponding proteins; Pr: protein; Underlined K in sequence is the cross-linked lysine.

*The crosslinked pair of residues were from two individual Ac-α-syn: ^14^N-Ac-α-syn and ^14^N-Ac-α-syn, ^14^N-Ac-α-syn and ^15^N-Ac-α-syn, ^15^N-Ac-α-syn and ^14^N-Ac-α-syn, or ^15^N-Ac-α-syn and ^15^N-Ac-α-syn. Detailed data processing procedure could be referred to Methods session.
